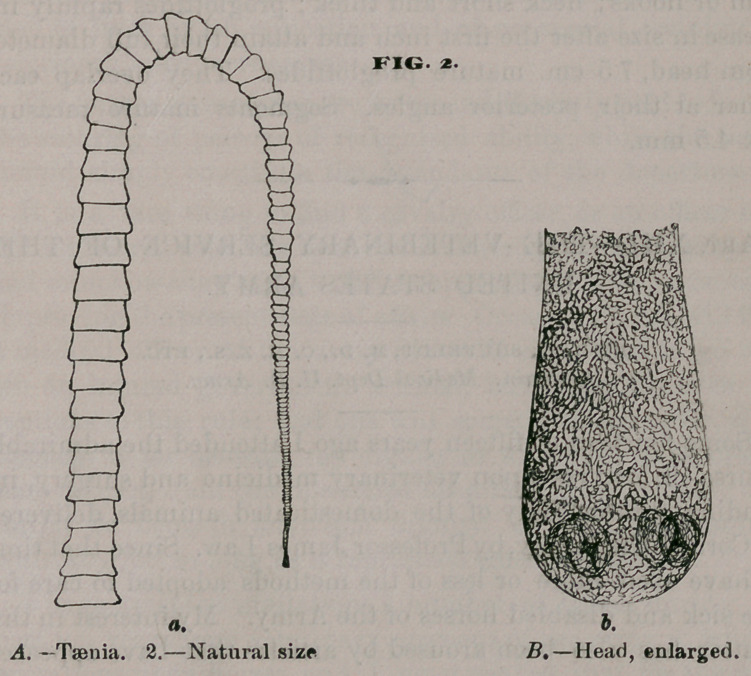# On Some Tæniæ of the Quadrumana

**Published:** 1887-04

**Authors:** William S. Gottheil

**Affiliations:** Lecturer on Pathology, American Veterinary College


### ART. XII.—ON SOME TÆNIÆ OF THE QUADRUMANA.

BY WILLIAM S. GOTTHEIL, M. D.,
*Lecturer on Pathology, American Veterinary College.*

Through the kindness of Dr. W. A. Conklin I had an opportunity to examine the bodies of several monkeys that died this spring in the Central Park Menagerie. A number of examples of two varieties of tæniæ were found. A careful search of the literature accessible to me in New York did not enable me to refer them positively to any of the described varieties. Beyond the general references in the text-books but one case, that of Studen, was found in the periodical records. Cobbold simply says that old-world monkeys rarely harbor full-grown tape worms—though cysticerci are not rare; and on the other hand that tæniæ are common in monkeys of the new world, but larval cestodes are seldom seen. He mentions *C. tennicollis, C. cellulosæ, C. pileatus, C. crispus, Echinococcus multiformis,* and *T. megastoma* and *T. rugosa* as the varieties that have been found. No one of these tæniæ correspond with the varieties figured below.

Professor Th. Studen, in the *Miltheilugen der Natur-forschend-Gesellsch-in Berne* for 1879, thus describes a tapeworm, two specimens of which were found in the duodenum of a chimpanzee two years old.

"Length, 100 mm., largest breadth at last segment 15 mm. Scolex small, club-shaped, four suckers, neither rostellum nor hooks. The segments rapidly get large from before backwards, so that the whole is tongued-shaped. Proglottides very short, 1 mm. by 3 mm. broad. Sexual orifices at sides, alternating and wart-shaped; they begin at the end of the first third of the whole. The entire colony was black, pigmented most deeply at the last segment; sexual openings white."

It is to be regretted that no drawing accompanies the account.

Of the tapeworm shown in Fig 1., two specimens were found, together with ten of the second variety, in a female Macaque

**FIG. I.**

*a.*

*b.*

*A.*—Tænia. 1.—Natural size. *B.*—Head, enlarged.

**FIG. 2.**

*a.*

*b.*

*A.* –Tænia. 2.—Natural size *B.*—Head, enlarged.

monkey, *Macacus cynomolgus.* The parasitic mass entirely occluded the duodenum, the stomach being full of food, and the intestine below entirely empty.

No. 1 (Fig. 1). Length 20 cm., breadth at largest segment 3.5 mm. Head globular, four suckers, no rostellum, and no hooks. Neck extremely fine and filamentous—2.5 cm. long. Proglottides slowly increase in size, greatest breadth being only attained 15 cm. from head. Sexual orifices at the sides—segments oblong, 3.5 mm. by 0.75 mm. Owing to the extreme delicacy of the head and neck but one specimen was obtained whole, and that one was almost destroyed in the attempt to mount it permanently after the drawing was made.

No. 2 (Fig. 2). Eleven specimens of this tapeworm were found, ten in the monkey previously referred to, and one, together with a considerable quantity of loose proglottides, in the intestines of a male weeping capuchin monkey—*Cebus capucinus.*

Length of largest specimen 15 cm. Breadth at largest segment 8 mm. Head large and clubbed, four suckers, no rostellum or hooks; neck short and thick; proglottides rapidly increase in size after the first inch and attain their full diameter from head, 7.5 cm. mature proglottides. They overlap each other at their posterior angles. Segments mature measure 8 x 4.5 mm.

## Figures and Tables

**FIG. 1. f1:**
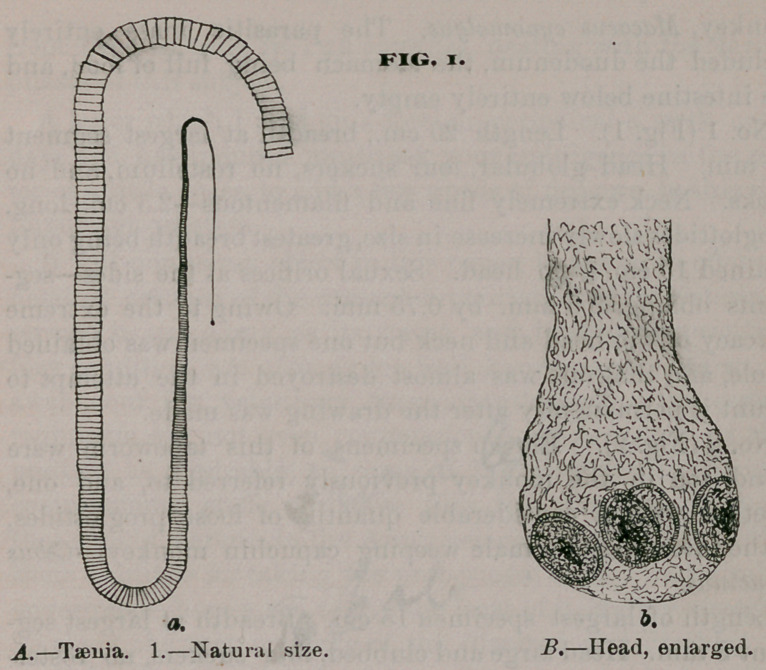


**FIG. 2. f2:**